# Ambiguity aversion, modern Bayesianism and small worlds

**DOI:** 10.12688/openreseurope.13196.1

**Published:** 2021-03-24

**Authors:** Nikitas Pittis, Phoebe Koundouri, Panagiotis Samartzis, Nikolaos Englezos, Andreas Papandreou

**Affiliations:** 1 Department of Banking and Financial Management, University of Piraeus, Piraeus, Greece; 2 School of Economics and ReSEES Laboratory, Athens University of Economics and Business; Sustainable Development Unit, Athena Research Center; World Academy of Arts and Science; UN SDSN-Europe, Athens, Greece; 3 School of Economics, National and Kapodistrian University of Athens, Athens, Greece

**Keywords:** uncertainty, ambiguity aversion, modern bayesianism, classical bayesianism, small worlds

## Abstract

The central question of this paper is whether a rational agent under uncertainty can exhibit ambiguity aversion (AA). The answer to this question depends on the way the agent forms her probabilistic beliefs: classical Bayesianism (CB) vs modern Bayesianism (MB). We revisit Schmeidler's coin-based example and show that a rational MB agent operating in the context of a "small world", cannot exhibit AA. Hence we argue that the motivation of AA based on Schmeidler's coin-based and Ellsberg's classic urn-based examples, is poor, since they correspond to cases of "small worlds". We also argue that MB, not only avoids AA, but also proves to be normatively superior to CB because an MB agent (i) avoids logical inconsistencies akin to the relation between her subjective probability and objective chance, (ii) resolves the problem of "old evidence" and (iii) allows psychological detachment from actual evidence, hence avoiding the problem of "cognitive dissonance". As far as AA is concerned, we claim that it may be thought of as a (potential) property of large worlds, because in such worlds MB is likely to be infeasible.

## 1 Introduction

Since 1961, when Ellsberg introduced the concept of ”ambiguity aversion” (AA) in the form of his well-known thought experiments (see Ellsberg, 1961), economists, psychologists and decision theorists have been trying to provide answers to the following two questions: (i) is AA empirically documented by means of formal experimentation and (ii) does AA violate the standard Bayesian conditions of rationality, or is it consistent with rationality when properly modified?

With respect to the first question, Machina & Siniscalchi (2014) offer an extensive survey of the empirical literature (including studies from the insurance and medical literature) that spans over a period of 50 years. The results of these experiments lend support to AA, although some results that point towards ”ambiguity neutral” or even ”ambiguity seeking” behavior do exist (see, for example, Viscusi & Chesson, 1999).

As far as the second question is concerned, the main argument in favor of AA being consistent with rational probabilistic beliefs is the following: AA implies that probabilistic beliefs are not sophisticated, in the sense that they cannot be represented by a unique *prior* probability measure. Is a probabilistically non-sophisticated agent necessarily irrational? The advocates of the argument that AA is consistent with rational probabilistic beliefs say no. Instead, they argue that the agent might possess so little (if any) information about the events she is interested in, that she is justifiably (and thus rationally) unable to form a unique prior distribution. As Gilboa & Schmeidler (1989) put it ”...the subject has too little information to form a prior. Hence (s)he considers a set of priors as possible.” (1989, pp. 142) ^1^.

Schmeidler (1989) is one of the earliest and best-known attempts to provide an axiomatization of AA. Schmeidler argues that at the heart of AA lies the following asymmetry: Consider an event space ℱ which contains the ”events of interest” for agent *X*. Assume that ℱ *_X_* ⊂ ℱ contains those events whose probabilities are known to *X*, whereas ${ℱ}_{X}^{\prime }$ ⊂ ℱ , ( ℱ *_X_* ∩ ${ℱ}_{X}^{\prime }$ = Ø, ℱ *_k_* ∪ ${ℱ}_{X}^{\prime }$ = ℱ ) contains the rest of events for which *X* has no information about. AA means that *X* favors betting on events in ℱ *_X_* than ${ℱ}_{X}^{\prime }$ . However, under the maintained assumption that *X*’s subjective probability of any event *D* ∈ ℱ is measured by *X*’s willingness to bet on *D*, AA implies that *X*’s degree of belief defined on ℱ violate the additivity axiom of probability theory, thus *X* is not probabilistically sophisticated.

What is the main reason for the violation of the additivity property in *X*’s degrees of belief, denoted by *P _X_*? The answer is that *X* has specific information, *I _S_*, which is relevant for some specific events in ℱ and irrelevant for all the rest. Is *I _S_* allowed to be used in the determination of the prior beliefs, or should these beliefs be based exclusively on the so-called ”background” information, *I _B_*?

How is *I _B_* defined? A universally accepted sharp definition of *I _B_* is hard to find. *I _B_* is usually meant to include the ”corpus of background knowledge” ( Easwaran, 2008) that describes the general characteristics of the chance set up in hand (e.g. there are *n* coins involved, each one is two-sided, successive tosses are independent, etc). More importantly, *I _B_* should include the full set of hypotheses *H* that describe the probabilistic properties of the phenomenon of interest, the full set of evidential sentences *E* that are relevant for *H* together with the entailment relationships between elements of *H* and elements of *E*. On the other hand, *I _B_* should not include any actual outcomes of the experiment or information about the objective chances (e.g. coin is fair). This information is specific information (or relevant evidence) and should be thought of as part of *I _S_*.

The question of whether to allow both *I _B_* *and I _S_* or only *I _B_* to affect the generation of the agent’s prior belief function is crucial for deciding whether AA is (or can be made) consistent with rationality. Hence the crucial question is: should the prior belief, *P _prior_*, depend only on *I _B_* (Option I) or on both *I _B_* *and* *I _S_* (Option II)? There are two approaches in the literature: one that claims that the distinction between *I _B_* *and* *I _S_* is not only useful but compelling, and the other which sees no point in distinguishing between the two. In the context of Option I, the prior probability (credence) is shaped by all the available information that the agent possesses at t, including both *I _B_* *and* *I _S_* In such a case, the evidential significance of *I _S_* is ”built into” *P _prior_*. Notationally, we may emphasize this, by replacing *P _prior_* with $P_{t}^{I_{B},I_{S}}.$ Following Meacham (2007), we refer to this view as ”classical Bayesianism” (CB). On the other hand, Option II, hereafter referred to as ”modern Bayesianism” (MB), amounts to the prior being determined without any influence from the specific information *I _S_*. In the case where the agent doesn’t possess any specific information at the time she wants to assign a probability to an event of interest, this condition is satisfied trivially. On the other hand, if she possesses specific information, this condition is satisfied if *X* employs a mode of counterfactual probabilistic reasoning. This type of reasoning can be described as a two-step procedure: i) The agent, at some point in time *t*, wants to assign a probability to an event of interest. She has at her disposal both background *I _B_* and specific information *I _S_*. ii) She travels back in time, at the start of her epistemic life, say *t*_0_, and forms her prior belief in light only of background information. Thus, specific information is not allowed to ”contaminate” the formation of her prior belief. Put differently, the prior probability function should be determined, once and for all, at the unique point in time *t*_0_, at which only *I _B_* is available. At *t*_0_, *I _S_* is contingent (hypothetical) rather than actual. Notationally, *P _prior_* may be replaced by $P_{0}^{I_{B}}.$

The central question of this paper is whether an agent with rational beliefs (i.e. an agent who satisfies Savage (1954) axioms) can exhibit AA. The answer to this question depends on the way the agent forms her probabilistic beliefs. On one hand, as already discussed, AA can be consistent with rationality for a CB agent. On the other hand, the main point of this paper is that an MB agent, operating in the context of ”small worlds” (more on this below), cannot exhibit AA. This brings about the question of whether MB is a normatively appealing hypothesis. To this end, we put forward three arguments in favor of MB, analyzed in detail in the section 3. In summary, we argue that an MB agent (as opposed to a CB one) (i) protects herself from falling into logical contradictions akin to the relation between her subjective probability and objective chance, (ii) fares better in cases of ”old evidence” and (iii) allows psychological detachment from actual evidence.

What about MB’s descriptive status? Is MB reasonably realistic as a model of actual behavior? Or is it the case that MB puts exceedingly high standards on the agent’s probabilistic reasoning abilities? To answer this question, we must first define the context within which the agent operates. To this end, we distinguish between ”small” and ”large” worlds, a distinction already made by Savage (1954). For the purposes of the present paper, a ”world” is defined to be the domain (language) ℒ of the agent’s probabilistic beliefs. This domain includes all the hypotheses of interest *H _i_*, all the relevant evidential statements *E _j_*, together with the corresponding (logical) entailment relations between *H _i_* and *E _j_*. A world is small if (a) the number of hypotheses in ℒ is small and (b) ℒ does not evolve over time. These two assumptions imply that the agent is able to conceive in advance all possible hypotheses concerning the matter of interest. In this paper we argue that MB is a reasonable hypothesis in small worlds.

On the contrary, MB is not likely to work in cases in which a) ℒ is exceedingly rich and b) ℒ changes over time due to the formation of new concepts (hypotheses) by the agent. With respect to the second case, imagine a situation in which the agent’s current domain, ℒ *_t_*, is richer than her domain ℒ _0_ at the beginning of her investigations, *t*_0_ (that is before any specific information *I _S_* is available). This may happen if between the time periods *t*_0_ and *t*, a new hypothesis *H _new_* was conceived by the agent. In such a case, *H _new_* is part of ℒ *_t_* but not of ℒ _0_. MB requires the agent to ”go back in time” in order to find herself in the epistemic state that corresponds to *t*_0_. Unfortunately, at this state, the agent is unable to find any probabilistic assignments to *H _new_* simply because *H _new_* was not included in ℒ _0_. In such a case, MB requires the agent to contemplate her probabilistic assignments not in the ”old” domain ℒ _0_ (which did not include *H _new_*) but in the ”new”, expanded domain ℒ *_t_*. However, this shift between domains means that the agent is forced to change all her probabilistic assignments made in the context of ℒ _0_. This is the point at which MB loses its normative and descriptive edge. As Bammer & Smithson (2012, pp 95) remark ”...changing the language (domain) in which items of evidence and hypotheses are expressed will typically change the confirmation relations between them.”

Do MB’s losses translate to CB’s gains? The answer is negative. Both MB and CB are equally vulnerable to the presence of large and evolving worlds because under those worlds, both MB and CB require frequent changes in prior probabilities $P_{0}^{I_{B}}.$ and $P_{t}^{I_{B},I_{S}}.$ Such changes invalidate many of the most interesting Bayesian theorems, including the celebrated ”washing out of priors ^2^” result.

It is worth emphasizing that standard Bayesianism, in either MB or CB form, does not allow the formation of new hypotheses after the prior probability function has been formed. Instead, it assumes that the agent is able to think in advance of all the possibilities in the hypothesis space; in short, the agent is assumed to be ”logically omniscient”. Logical omniscience is considered by many critics as the Achilles heel of Bayesianism: it implies an all-knowing agent who is able to track down all the logical implications in ℒ . Obviously, the extent to which such an assumption is unrealistic depends on the degree of richness of the underlying domain ℒ . For simple domains, it is reasonable to assume that the agent can comprehend the few logical entailments that these domains contain, which renders standard Bayesianism realistic in small worlds.

The main points of the paper are the following: (i) An MB agent operating in small worlds does not exhibit AA, (ii) AA may be thought of as a (potential) property of large worlds, because in such worlds MB is likely to be infeasible, and (iii) The motivation of AA in the form of examples such as Ellsberg’s classic urn-based or Schmeidler’s coin-based ones is clearly poor, since these examples are textbook cases of small worlds (or elementary domains), in which the normatively preferable MB strategy is attainable.

The paper is organized as follows: section 2 describes Schmeidler’s two-coin thought experiment that is supposed to produce AA. It also defines formally two of the central concepts of this paper, namely MB and CB. Section 3 is an analytic discussion of the arguments for preferring MB over CB. Section 4 provides the main objection to MB, namely logical omniscience. It also elaborates on the connection between MB and small worlds and the efficiency of counterfactual probabilistic reasoning in such worlds. Section 5 demonstrates formally the absence of AA under MB reasoning within Schmeidler’s two-coin example. Section 6 concludes the paper.

## 2 Schmeidler’s two-coin example and modern versus classical Bayesianism

Schmeidler (1989) uses the following coin example, which aims at conveying the same message with Ellsberg’s two-urn paradox (1961). Assume that the agent *X* considers two coins A and B. *X* knows with certainty that the probability of ”heads” for A ( *H _A_*) is 0.5. On the other hand, she has no such information about B. The two coins are about to be tossed and *X* has the option to bet either on A-related events or B-related events. Specifically, she may choose bet A in which she earns 1$ if the event


$$D_{H}^{A}=\left\{\mathrm{coinAcomesupheads}\right\}=\left\{H_{A}H_{B},H_{A}T_{B}\right\}$$

occurs and loses 1$ if the following event materializes


$$D_{T}^{A}=\left\{\mathrm{coinAcomesuptails}\right\}=\left\{T_{A}H_{B},T_{A}T_{B}\right\}.$$

Alternatively, she is offered the option to enter bet B which gives her 1$ if the event


$$D_{H}^{B}=\left\{\mathrm{coinBcomesupheads}\right\}=\left\{H_{A}H_{B},T_{A}H_{B}\right\}$$

materializes and produces a loss of 1$ if the event


$$D_{T}^{B}=\left\{\mathrm{coinBcomesuptails}\right\}=\left\{H_{A}T_{B},T_{A}T_{B}\right\}$$

takes place. Obviously,


$$D_{H}^{A},D_{T}^{A}\in {ℱ}_{X}$$

whereas


$$D_{H}^{B},D_{T}^{B}\in {ℱ}_{X}^{\prime }.$$

Indeed, the agent knows that


P(DA=P(DTA)=0.5

although she is not informed about the probabilities P(DB and P(DB. However, although she does not know the exact values of P(DB and P(DB she feels more willing to bet on $D_{H}^{A}$ than $D_{H}^{B}$ and on $D_{T}^{A}$ than $D_{T}^{B}$ , i.e.


$$P(D_{H}^{B})<P(D_{H}^{A})$$

and


$$P(D_{T}^{B})<P(D_{T}^{A}).$$

It is easy to show that such a *P* is non-additive. Indeed,


$$D_{H}^{B}\cap D_{T}^{B}=Ø$$

and


$$D_{H}^{B}\cup D_{T}^{B}=\Omega$$

where Ω is the relevant sample space, namely


$$\Omega =\left\{H_{A}H_{B},H_{A}T_{B},T_{A}H_{B},T_{A}T_{B}\right\}.$$

Assuming that *P* is additive,


$$1=P(D_{H}^{B}\cup D_{T}^{B})=P(D_{H}^{B})+P(D_{T}^{B})<1$$

which is a contradiction.

What causes the violation of the additivity property in *P*? It is not merely the presence of asymmetric information, but rather the fact that the agent allowed *I _S_* to affect directly her probabilistic beliefs, instead of utilizing (as she should) *I _S_* indirectly by conditionalization. But in order to be able to conditionalize on *I _S_*, a pre-existing vehicle is required, namely a probability function, prior to the acquisition of *I _S_*. Put differently, the agent should form her probabilistic beliefs by employing the MB counterfactual strategy outlined in the introduction.

Let us now define the concepts of MB and CB more formally. To this end, assume an agent, who started her investigations at time *t*, having both *I _B_* and *I _S_* at her disposal. The agent is interested in updating her beliefs about the proposition/event *A* in the light of new evidence *E* obtained at *t* + 1. A CB agent carries out this task as follows:

CB:

$$P_{t\text{+1,}I_{B},I_{S},E}(A)\;=\;P_{t}^{I_{B},\;I_{S}}(A\;|\;E).\;(1)$$

On the other hand, the MB strategy advises the agent to pursue the following two-step procedure: First, ”travel back” to your epistemic history and identify point, *t*_0_, at which *I _S_* was not yet available (although it was conceivable). Second, once this point was found, evaluate the prior that you would have formed at this point (counterfactual or hypothetical prior) and use it from this point onwards as your actual prior.

MB:

$$P_{t\text{+1,}I_{B},I_{S},E}(A)\;=\;P_{0}^{I_{B}}(A\;|\;I_{S},\;E).\;(2)$$

Easwaran (2008) refers to $P_{0}^{I_{B}}$ ”as expressing counterfactual possibilities of what she (the agent) imagines she would believe if she didn’t have some of the information (in our case, *I _S_*) she does now.” (2008, pp.147). In a similar fashion, Meacham (2008) explains the epistemic role that $P_{0}^{I_{B}}$ is supposed to play: ”A rational subject’s credences are fixed by her hypothetical priors and her total evidence. A subject’s credences are represented by a dynamic probability function, a function that changes with her evidence. A subject’s hypothetical priors are represented by a static probability function, a function that encodes her disposition to respond to evidence. (Hypothetical priors are called ‘priors’ because they can be thought of as a rational subject’s original credences in possibilities, prior to the receipt of any evidence, and ‘hypothetical’ because it is unlikely that one ever was in such a state.)” (2008, emphasis added). It is worth emphasizing that despite the term ”modern”, MB is not a form of Bayesianism that was put forward only recently. Indeed, several authors from the 1970s entertained the idea of ”hypothetical priors”, that is, ”priors without evidence” ^3^.

What should the basic probability concept be in the development of rational decision theory? Prior or Current Probability? To answer this question we must think of what personal probabilities should reflect if they were to be rational: the person’s permanent dispositions for forming beliefs (on the basis of the available evidence) or merely her momentary inclinations at some given point in time?

Rudolph Carnap argues strongly in favor of the first option. Indeed, his monumental work on inductive logic ( Carnap, 1950) is based on the concept of hypothetical or counterfactual initial credence function that can be ascribed to the agent X, before the collection of any evidence. More specifically, Carnap (1962) considers a sequence of data *E*_1_, *E*_2_, ..., *E _n_* obtained by the agent X up to the present time *T _n_* (in Carnap’s notation). He also defines *K _n_* to be the ”total observational knowledge” of X at *T _n_*, that is

$$K_{n}\;=\;\cap_{i=1}^{n}\;E_{i}.\;(3)$$

Then Carnap contemplates X’s epistemic state at time *T*_0_ in which X possesses no observational knowledge at all: ”Now consider the sequence of X’s credence functions. In the case of a human being we would hesitate to ascribe to him a credence function at a very early time point, before his abilities of reason and deliberate action are sufficiently developed. But again we disregard this difficulty by thinking either of an idealized human baby or of a robot. We ascribe to him a credence function *Cr*_1_ for the time point *T*_1_; *Cr*_1_ represents *X ^′^s* personal probabilities based upon the datum *E*_1_ as his only experience. *Going even one step further*, let us ascribe to him an initial credence function *Cr*_0_ for the time point *T*_0_ *before he obtains his first datum* *E*_1_. Any later function *Cr _n_* for a time point *T _n_* is uniquely determined by *Cr*_0_ and *K _n_* : For any *H,*

$$Cr_{n}(H)\;=\;Cr_{0}^{\prime }(H\;|\;K_{n})\;,\;(4)$$

where ${\mathrm{Cr}}_{0}^{\prime }$ is the conditional function based on *Cr*_0_” (1962, pp. 310, emphasis added). Is *Cr*_0_ actual or counterfactual? Carnap explicitly allows the initial credence function to be hypothetical: He first raises the question: ”How can we understand the function *Cr*_0_?” The answer to this question depends on whether the agent X is a robot or a human being. In the first case, *Cr*_0_ is robot’s actual credence function at *T*_0_. With respect to the second (more relevant case) Carnap’s answer is as follows: ”In the case of a human being X, suppose that we find at the time *T _n_* his credence function *Cr _n_*. Then we can, under suitable conditions, reconstruct a sequence *E*_1_, *E*_2_, ..., *E _n_*, the proposition *K _n_*, and a function *Cr*_0_ such that (a) *E*_1_, *E*_2_, ..., *E _n_* are possible observation data, (b) *K _n_* is defined by ( 3), (c) *Cr*_0_ satisfies all requirements of rationality for initial credence functions, (d) the application of ( 4) to the assumed function *Cr*_0_ and *K _n_* would lead to the ascertained function *Cr _n_.* We do not assert that X *actually experienced the data E*_1_ *, E*_2_ *, ..., E _n_*, and that he *actually had the initial credence function Cr*_0_, but merely that under idealized conditions, his function *Cr _n_* could have evolved from *Cr*_0_ by the effect of the data *E*_1_ *, E*_2_ *, ..., E _n_.*” (1962, pp. 310, emphasis added)

Why does Carnap identify the initial credence function *Cr*_0_, instead of the current credence function *Cr _n_*, as the basic concept upon which (any) rational decision theory must be built? The answer to this question is based on the distinction between the two competing concepts that drive a person’s (say X) degrees of belief mentioned above, namely: (i) X’s momentary inclination at time T and (ii) X’s permanent disposition to believe. Carnap argues that what we are interested in, in developing rational decision theory is the second concept, that is a ”trait” of X’s ”underlying permanent intellectual character”. This disposition is best reflected on X’s initial credence function, that is X’s degrees of belief prior to the acquisition of any evidence. Any change in X’s beliefs, due to the emergence of new evidence, *E*, should be based on *Cr*_0_( *· | E*) *.* Such a conditionalization ensures that X’s current credences will also be driven by X’s permanent disposition for forming beliefs on the basis of the received evidence. To this end, Carnap argues as follows: ”When we wish to judge the morality of a person, we do not simply look at some of his acts, we study rather his character, the system of his moral values, which is part of his utility function. Single acts without knowledge of motives give little basis for a judgement. Similarly, if *we wish to judge the rationality of a person’s beliefs, we should not simply look at his present beliefs. Beliefs without knowledge of the evidence out of which they arose tell us little. We must rather study the way in which the person forms his beliefs on the basis of evidence.* In other words, we should study his credibility function, not simply his present credence function.” (1962, pp. 312, emphasis added).

The distinction between actual versus hypothetical priors in the context of Schmeidler’s two-coin example may be described as follows: Assume that at time *t*, in which the desire for a specific investigation or bet emerged (for example the time at which the agent decided to bet on $D_{H}^{A}$ ), specific information *I _S_* already existed (for example, the coin A has already been flipped five times, with the results being *H _A_, H _A_, T _A_, H _A_, T _A_*). What is the agent advised to do in this case? Should she form her priors by taking *I _S_* into account? Or should she ”travel back in time” and ask herself ”what would have my priors been at time *t*_0_ when *I _S_* was not available?” MB responds negatively to the first question and positively to the second.

Based on the above MB recommendation, the following pressing question presents itself: Why should the agent take the burdensome and imagination-stretching counterfactual route ( 2) in forming her current beliefs, while the alternative actual (and intuitively appealing) course ( 1) is readily available? Put differently, why should we require the agent to ponder her subjective probabilities under an epistemic state which is not her actual state of knowledge but rather a counterfactual one? There are at least three reasons that compel an agent to follow the counterfactual path: These reasons are analyzed in detail in the next Section. 

## 3 Arguments for MB

### 3.1 MB and the chance-credence relationship: avoiding inconsistencies

Principal Principle (PP), originally suggested by David Lewis (1973), aims at capturing the following intuitive idea: If the agent X comes to know (with certainty) that the objective probability of the event (proposition) A, *Ch*( *A*) *,* is p and does not possess any inadmissible evidence for A ^4^, then X’s subjective probability of A must be set equal to p. The following argument discusses a special case, under which MB is consistent with PP, whereas CB is not consistent with PP.

How to describe formally the PP? One option is to use the current credence function $P_{t}^{I_{B},I_{S}}$ , which gives:


$$P_{t}^{I_{B},I_{S}}(A|<Ch(A)=p>,E)=p,\;(5)$$

where *E* is admissible evidence obtained at *t* + 1 *.*

The first version of PP, given by ( 5), may be interpreted in the light of CB, as follows: The agent X, being at time t, has at her disposal *I _B_* and *I _S_.* Being a CB, the agent uses both these items of information to generate her ”unconditional” probability $P_{t}^{I_{B},I_{S}}$ ( *A*) at t. If she acquires a further item of information *E* then her new updated probability of A would be


$$P_{t+1,I_{B},I_{S},E}(A)=P_{t}^{I_{B},I_{S}}(A|E).$$

However, if in addition to *E* the agent learned that the chance of A is *p*, then the latter piece of information would dominate the formation of her beliefs, in the sense that *< Ch*( *A*) = *p >* screens-off any admissible evidence, E, for A, thus yielding ( 5). Hence, her new (updated) probability of A is given by,


$$P_{t+1,I_{B},I_{S},E,<\;Ch(A)=p>}(A)=P_{t}^{I_{B},I_{S}}(A|<Ch(A)=p>,E)=p.\;(6)$$

A second option for the agent is to operate as an MB, thus treating *I _S_* not as actual but rather as counterfactual. As a result, she employs the initial prior probability function $P_{0}^{I_{B}}$ , instead of the current one, thus yielding the following

version of PP:


$$P_{0}^{I_{B}}(A|<Ch(A)=p>,E,I_{s})=p\;\mathrm{if}\;E\;(\mathrm{and}\;I_{s})\;\mathrm{is admissible}.\;(7)$$

What is the restriction that ( 7) imposes on X’s current credences? Put differently, assume X is currently at time *t* + 1, in which she has come to know *I _S_,* *< Ch*( *A*) = *p >* and *E.* MB’s condition ( 2) implies the following relation:


$$P_{t+1,I_{B},I_{S},E,<\;Ch(A)=p>}(A)=P_{0}^{I_{B}}(A|<Ch(A)=p>,E,I_{s})=p.\;(8)$$

Comparing ( 6) with ( 8), we form the impression that it makes no difference to the agent’s updated probability of A, whether she acts as a CB or MB. However, Meacham (2007) argues that this is not the case. He argues that ( 5), as opposed to ( 7), leads to contradictions. To understand why, we must first emphasize the difference that the role that *I _S_* plays in ( 5) versus ( 7): On one hand, under the MB formulation ( 7) of PP, *I _S_* is conditioning information and therefore, we must examine if it is admissible or not. Now, let us consider the special case in which A is included in *I _S_* at time *t.* In such a case, *I _S_* becomes automatically inadmissible and therefore, PP becomes non-applicable and the agent’s probability should be set equal to one.

On the other hand, *I _S_* is not conditioning information; it is rather a direct determinant of the agent’s current probability function $P_{t}^{I_{B},I_{S}}.$ As such, admissibility is not relevant. Therefore, the agent cannot condition on A since it has already determined her probability function. Hence, ( 5) dictates that the agent’s subjective probability of A should be equal to *p <* 1. But on the other hand, this probability should be equal to one, since A is known to the agent. Hence, we have run into the following contradiction:


$$1=P_{t}^{I_{B},I_{S}}(A|<Ch(A)=p>,E)=p\neq 1$$

Obviously the extent to which the prior probability is preferable to the current probability for both formal and conceptual reasons, determines the extent to which MB is preferable to CB.

### 3.2 MB and the problem of old evidence

The ”problem of old evidence” concerns the role of evidence, *E*, that was already known (hence, old) at the time of the formation of a scientific theory ( *T*) for the confirmation (or not) of *T*. The problem was first presented by Glymour (1980) who described it as follows: ”Scientists commonly argue for their theories from evidence known long before the theories were introduced. Copernicus argued for his theory using observations made over the course of millennia.... Newton argued for universal gravitation using Kepler’s second and third laws, established before the Principia was published. The argument that Einstein gave in 1915 for his gravitational field equations was that they explained the anomalous advance of the perihelion of Mercury, established more than half a century earlier.... Old evidence can in fact confirm new theory, but according to Bayesian kinematics it cannot. For let us suppose that evidence *e* ( *E* in our notation) is known before theory *T* is introduced at time t. Because *e* is known at t, *P _t_* ( *e*) = 1. Further, because *P _t_* ( *e*) = 1, the likelihood of *e* given *T*, *P _t_* ( *e|T*), is also 1. We then have:


$$P_{t}(T|e)=\frac{P_{t}(T)P_{t}(e|T)}{P_{t}(e)}=P_{t}(T)$$

The conditional probability of *T* on *e* is therefore the same as the prior probability of *T* : *e* cannot constitute evidence for *T*.... None of the Bayesian mechanisms apply, and if we are strictly limited to them, we have the absurdity that old evidence cannot confirm a new theory.” (1980, pp. 85–86). The previous quotation highlights a conflict between Bayesian theory of confirmation and the standard scientific practice when it comes to the use of *E*. Specifically, *E*, under the standard scientific practice, can in fact confirm new theory, but according to Bayesian theory it cannot.

Bayesian philosophers have responded to the problem of old evidence in (mainly) two different (and incompatible) ways. The first response, put forward by Garber (1983), Niiniluoto (1983) and Jeffrey (1983) goes as follows: What makes the scientist to increase her confidence in *T*, is not *E* per se, since this information was already known to her at the time she invented her theory. Rather it was the realization of the *logical entailment relationship*


T⊢E(9)

that made the scientist to increase her confidence in *T.* For example, ”Einstein discovered only after writing down the equations of General Relativity that they entailed the anomalous perihelion advance of Mercury.” ( Howson, 1991, pp. 553). Hence, the increase in the agent’s degree of belief in *T* comes from conditionalization on the entailment relationship ( 9) itself rather than conditionalization on just *E*. The proposition ( 9) at *t*_0_ was uncertain, in the sense that the agent had not established/proved the validity of the postulated entailment relationship, i.e.


$$P_{0}(T⊢E)<1,$$

in which case the realization of ( 9) at t results in an increase in probability of *T.*

Note that this line of defense is based on the assumption that the agent conditionalizes on the entailment ( 9), which in turn implies that at *t*_0_, the agent had already assigned a prior probability to this proposition, i.e *P*_0_( *T⊢E*) = *p*, with *p <* 1 *.* This means that at *t*_0_ the agent was not aware of the truth of ( 9) (which truth she discovered only later) which in turn implies that at *t*_0_ the agent had assign a probability less than one to a logical truth. This, however, means that *P*_0_ is not coherent. Hence, this line of defense against the problem of old evidence leads to another problem (potentially more serious) which is incoherence of the agent’s probability function.

The second argument for solving the problem of old evidence is more in the spirit of MB since it is based on the following counterfactual ”what-if” strategy. Garber (1983) portrays this strategy as follows: ”One obvious response might begin with the observation that *if one had not known the evidence in question*, then its discovery *would have increased one’s degrees of belief* in the hypothesis in question ( *H*). That is, in the circumstances in which *E* really does confirm *H*, if it had been the case that *P*( *E*) *<* 1, then it would also have been the case that *P*( *H|E*) *> P*( *H*).” (1983, pp. 103, emphasis added). The solution outlined by Garber, suggests that the agent should not evaluate her probabilities relative to *I* = *I _B_* ∪ *I _S_* but only relative to *I _B_.* This is exactly the proposal offered by Howson (1991) in his attempt to solve the problem of old evidence. Indeed, he explicitly states that the probabilities ”should always be relativized to *K* − { *E*}” (1991, pp. 548, with *K* and *E* in Howson’s notation corresponding to *I _B_* and *I _B_*, respectively) ^5^. But, as analyzed in the introduction, this is exactly the point at which MB and CB differ. Hence, choosing MB over CB might be motivated by the fact that the first strategy fares better than the second in cases of ”old evidence”.

### 3.3 MB and cognitive dissonance

Let us now turn our attention to the third reason for preferring the counterfactual MB strategy over the actual CB one. This reason is akin to the possibility that the agent evaluates the entailment relationships between theoretical and evidential statements in a more unbiased way when the evidence *I _S_* is hypothetical than when it is actual. The psychological finding of treating hypothetical and actual evidence differently, relates to the theory of cognitive dissonance, used by Albert Hirschman (1965) to describe attitude changes toward modernization in the course of development. More generally, cognitive dissonance stems from the fact that persons who have made decisions, tend to discard information that would suggest such decisions are wrong. As Akerlof & Dickens (2012) put it, ”Using Bayesian decision rules, agents’ estimates of the state of the world is only influenced by the information available to them and their preferences over states of the world, but these estimates are independent of their preferences for beliefs per se. The cognitive dissonance model not only predicts systematic differences in interpretation of given information but also systematic differences in receptivity to new information according to preferences.”

Under our setup, the probability that the agent assigns to the hypothesis *H _A_*: ”I shall live for at least another five years” conditional on the actual event *B*: ”I am diagnosed with aggressive lung cancer” is likely to be bigger than the probability that she would have assigned to *H _A_* if the event *B* were not actual but just a member of a set of many hypothetical events including the event non- *B*. In this example, the actual knowledge of the unfortunate event *B* at time t (now) is likely to create psychological pressure to distort the partial entailment relationship between *H _A_* and *B* that the agent was willing to accept at *t*_0_ *,* thus yielding


$$P_{t,B}(H_{A})>P_{0}(H_{A}|B).$$

This argument suggests that the subjective evaluation of probabilities should be made, as Longino (1979, pp. 35) puts it, ”in rational, cool, moments”. As already mentioned, such a ”cool” and ”impartial” agent is an MB agent. In other words, an MB agent will never succumb to cognitive dissonance.

## 4 Objections to MB: logical omniscience

The arguments presented above suggest that MB is the optimal strategy that must be followed by an agent who is in the process of forming her subjective probability function at *t* (now). For reasons of clarity, this strategy is summarized below. The agent is currently at time *t*, knowing both *I _B_* and *I _S_.* The agent is advised to ”pretend” that she does not know *I _S_* and perform the following thought experiment: go back in time at period *t*_0_ in which only *I _B_* was available. At *t*_0_ *,* the background information *I _B_* contains the full set of hypotheses **H** that the agent actually entertained at *t*_0_ *,* the full set of evidential sentences **E** that the agent conceived as possible at *t*_0_, (every conceivable possible course events after *t*_0_) as well as all the entailment relationships between elements of **H** and elements of **E** (e.g. *H _i_* ⊢ *E _j_*). More specifically, each member of **E** describes the observations that have not been actually made at *t*_0_ but are considered by the agent at *t*_0_ as possible to make at the future period *t*. At *t*_0_ the agent’s subjective probability function *P*_0_ was defined over the field of propositions *L* (which includes **H** and **E**). Assume that at t, the particular element *E* ∈ **E** is realized, which implies that the agent at t has this specific information, i.e. *I _S_ ≡ E.* According to the MB counterfactual strategy, the agent’s new probability function, *P _t_,* at t should be her old probability function *P*_0_ conditional on *E*. As a result, her unconditional new probability for the uncertain event *A* (that will occur at t+1) should be set equal to the conditional probability *P*_0_( *A | E*) rather than being determined ”from scratch” as *P _t,E_* ( *A*) *.*

Is the above counterfactual strategy without any problems? The answer is negative. The main objections raised against this strategy revolve around the concept of logical omniscience (or its lack thereof). Specifically, observe that in the description of the counterfactual strategy presented above, we defined **H** and **E** as the sets of theoretical and evidential statements, respectively, *conceived by the agent* at *t*_0_ *.* However, Bayesianism assumes something significantly more than that. It assumes that the agent knows *all* the possible hypotheses, **H** ^*^ *all* the possible evidential statements **E** ^*^ and *all* the possible entailment relationships between **H** ^*^ and **E** ^*^; in other words the agent is assumed to be logically omniscient and her prior probability function, $P_{0}^{*}$ , is defined over the field ${ℒ}^{*}$ of *all propositions,* which is (infinitely) richer than ℒ . Why does Bayesianism require such an extreme condition? The answer is to ensure coherence of $P_{0}^{*}$ . Specifically, if we wish (as Bayesianists do) to ensure that $P_{0}^{*}$ is a proper probability measure then we must establish that to each logical consequence *p* ⊢ *q* the agent assigns probability equal to one, that is:


$$\forall (p⊢q),P_{0}^{*}(p⊢q)=1.$$

This is because if *p* logically entails *q,* it does so in every possible state of the world. Hence, it appears that coherence requires that the agent is capable of tracking all logical consequences at *t*_0_. In other words, the agent is not allowed to be unaware even of a single logical truth that exists within the underlying domain ℒ . As will be shown below only under this extreme form of logical omniscience the aforementioned MB counterfactual strategy is expected to work under all epistemic states that the agent may entertain at *t*.

### 4.1 Local vs. global Bayesianism

Logical omniscience of the form described above is a condition that is usually met within the variant of Bayesianism, known as global Bayesianism (GB). Garber (1983) defined GB as follows: ”One popular conception of the Bayesian enterprise is what I shall call global Bayesianism. On this conception, what the Bayesian is trying to do is build a global learning machine, a scientific robot that will digest all of the information we feed it and churn out appropriate degrees of belief. On this model, the choice of a language (domain) over which to define one’s probability function is as important as the constraints that one imposes on that function and its evolution. On this model, *the appropriate language to building into the scientific robot is the ideal language of science, a maximally fine grained language ℒ , capable of expressing all possible hypotheses, all possible evidence, capable of doing logic, mathematics*, etc. In short, ℒ must be capable, in principle, of saying anything we might ever find a need to say in science.” (1983, pp. 110 emphasis added).

In what respect, does the MB strategy suffer in the case of an agent whose prior probability function is *P*_0_ rather than $P_{0}^{*}$ ? This question is equivalent to the following one: ”Why is GB not realistic?”. The problem is the following: Assume that at *t* the agent comes across a piece of evidence E ^*^ that she had not conceived of as possible at *t*_0_ *.* Assume that this piece of evidence is the agent’s specific information ( *I _S_*) obtained at t. According to MB, the agent should go back to *t*_0_ and conditionalize on E ^*^ in order to generate her new probability function at *t.* However, an unpleasant surprise awaits the agent: At *t*_0_ there is no probabilistic assignment on E ^*^ simply because E ^*^ was not in the domain of *P*_0_ at *t*_0_ *.* To make things even worse, assume that E ^*^ was obtained at t because a new hypothesis, H ^*^, was put forward at *t* which motivated the observations (or experiments) that led to E ^*^. Obviously, H ^*^ was not in the domain of *P*_0_ either. For example, consider as E ^*^ and H ^*^ the ”deflection of light by the sun” and ”Einstein’s general relativity theory”, respectively. If a physicist of the late nineteenth century was asked to assign his personal probability to the event that the light is deflected by the sun, he would have been caught by surprise. His domain, ℒ at *t*_0_ was simply not rich enough to accommodate either E ^*^ or H ^*^ (let alone the entailment H ^*^ ⊢ E ^*^). In such a case, counterfactual conditionalization seems impossible and the whole MB strategy runs into trouble.

The preceding analysis suggests that the MB counterfactual strategy is unrealistic to the same extent as GB, (as a description of the epistemic state of real people) is. If the MB strategy is unrealistic within the GB framework, then is it possible that there is another framework, local Bayesianism (LB), within which the MB strategy is likely to work? Before we answer this question, let us first describe, following Garber (1983) how LB might be defined: ”Typically when scientists or decision makers apply Bayesian methods to the clarification of inferential problems, they do so in a much more restricted scope than global Bayesianism suggests, *dealing only with the sentences and degrees of belief that they are actually concerned with, those that pertain to the problem at hand*. This suggests a different way of thinking about the Bayesian learning model, what one might call *local Bayesianism*. On this model, the Bayesian does not see himself as trying to build a global learning machine, or a scientific robot. Rather, the goal is to build a hand-held calculator, as it were, a tool to help the scientist or decision maker with particular inferential problems. On this view, the Bayesian framework provides a general formal structure in which one can set up a wide variety of different inferential problems. In order to apply it in some particular situation, *we enter in only what we need to deal with in the context of the problem at hand, i.e., the particular sentences with which we are concerned, and the beliefs (prior probabilities) we have with respect to those sentences*.” (1983, pp. 111, emphasis added).

Formally, assume that the investigator is interested in the set of specific hypotheses (theoretical propositions) *H _i_, i* = 1 *,* 2 *, ..., n* that cover the full set of possibilities for the problem at hand (e.g. the coin is fair, the coin is biased towards H, the coin is biased towards T, etc). Related to the aforementioned theoretical propositions are the set of evidential propositions *E _j_ , j* = 1 *,* 2 *, ..., m* which are relevant for *H _i_* (e.g the outcome of the next toss is H, the outcomes of the next two tosses are H,T etc). No other proposition enters the agent’s ”problem over relative domain” ℒ that is relevant for the specific problem at hand. More specifically, Garber (1983, pp. 111) defines ℒ to be ”just the truth-functional closure” of the *H _i_*, *E _j_* and propositions of the logical entailment between *H _i_* and *E _j_*, that is propositions of the form ” *H _i_* ⊢ *E _j_*”. As a result, the agent’s prior probability function is defined over the ”local” more modest domain ℒ rather than the ”global” ideal domain ${ℒ}^{*}$ . This in turn implies that it is much easier for the agent to track all logical consequences ” *H _i_* ⊢ *E _j_*” (for some *i* and *j*) within ℒ than within ${ℒ}^{*}$ , thus making local Bayesianism more realistic than global Bayesianism (Bayesianism with a human face, as Jeffrey (1983) puts it).

### 4.2 Small vs. large worlds

The description presented above makes clear that LB refers to well-defined, small-scale cases (characterized by a small number of hypotheses, evidential propositions and logical relationships) which are usually referred to as ”small worlds”. In such a world, it is quite plausible to assume that the agent can consider all the possibilities (hypotheses, evidential propositions and their entailment relations) that are relevant for the problem at hand at *t*_0_. In such a case, no surprises await the agent at *t*, in the sense that his epistemic state at *t* is identical to that at *t*_0_ *.* Binmore (2009) defines a small world as one ”within which all potential surprises have been predicted and evaluated in advance of their occurrence.” (2009, pp 8).

What are the implications of small worlds for the effectiveness of the MB counterfactual strategy? Binmore (2009) comments on this question as follows: ”Only in a small world, in which you can always *look before you leap*, it is possible to consider everything that might be relevant to the decisions you take.” (2009, pp. 139 emphasis added). Indeed the ”look before you leap” proverb is attributed to Savage who used it as antithetical to ”cross that bridge when you come to it” that referred to the so called ”large worlds”. It is worth mentioning that Savage himself made quite clear that his own conception of subjective probability together with its axiomatization is relevant only for small worlds. This is because Savage’s framework is essentially static in the sense that it does not allow for the so-called ”concept formation”, that is the formation of a new hypothesis or a new idea sometime in the future. Commenting on Savage’s approach, Suppes (1966) observes: ”The important thing I wish to emphasize is that the theory provides no place for the decision-maker to acquire a new concept on the basis of new information received. The theory is static in the sense that it is assumed that the decision-maker has a fixed conceptual apparatus available to him throughout time.” (1966, pp. 21). Savage himself acknowledged the fact that the static nature of his theory makes it inapplicable in the case of large evolving worlds by referring to such an extension as ”ridiculous” and ”preposterous”.

## 5 Small worlds, counterfactual strategy and ambiguity aversion

The aforementioned discussion may be summarized as follows: MB and CB refer to the agent’s strategy or course of action in forming her probability function at GB and LB refer to the agent’s epistemic status at *t*_0_ as reflected in the contents of her background information *I _B_* or her domain, ( ℒ or ${ℒ}^{*}$ ) *.* Whether MB or CB is appropriate depends on which epistemic status, GB or LB characterizes the agent. Obviously there are four combinations for the agent to consider in her attempts to solve the problem of forming her subjective probability at *t ^*′*^*:

(i) MB-GB: ideal but unrealistic (infeasible) solution.
(ii) MB-LB: the appropriate solution in small worlds.
(iii) CB-GB: a feasible solution in large worlds but with the kind of problems analyzed in section 3.
(iv) CB-LB: inappropriate or inefficient solution.

How does the above classification bear upon AA? The preceding discussion has made clear that the fact that the agent allowed *I _S_* to affect directly her probabilistic beliefs, is only a necessary condition for AA. A further condition is that the problem at hand falls into the category of large worlds. Let us clarify this argument, which forms our basic thesis in this paper. Assume that the agent at time *t* has information, *I _S_*, about the objective probabilities of the events in ℱ *_k_*, but she lacks similar information about the events in ${ℱ}_{k}^{\prime }$ . If the world is small, the agent can go back in time at *t*_0_ *,* ignore *I _S_* and assign probabilities in ℱ ∪ ${ℱ}_{k}^{\prime }$ in an information-symmetrical way. Once her prior probability is thus determined (counterfactually at *t*_0_), the agent is allowed to bring *I _S_* back to the picture by conditionalizing on it. In such a case, the only way for the agent to produce AA is to deviate at *t* from her probabilistic commitments at *t*_0_, that is by exhibiting dynamic inconsistency. All the cases motivating AA that have appeared in the literature (including the original Ellsber’s paradox as well as Schmeidler’s two coin example) are ”textbook cases” of small worlds. Hence, although we do not claim that AA cannot arise in large worlds, (because in such a context the counterfactual MB strategy is unrealistic) we do argue that AA has been poorly motivated!

## 6 Schmeidler’s two-coin example revisited

Let us now revisit Schmeidler’s two-coin example discussed in the introduction in light of the analysis presented in section 4. First describe the agent’s epistemic background at *t*_0_ *.* For simplicity, we assume that concerning coin A, the agent knows with certainty that only one of the following three hypotheses is true:


$${ℋ}_{1}^{A}=\left\{\mathrm{CoinAisfair}\right\}$$

$${ℋ}_{2}^{A}=\left\{\mathrm{CoinAfavorsH}\right\}$$

$${ℋ}_{3}^{A}=\left\{\mathrm{CoinAfavorsT}\right\}.$$

To make things even simpler assume that in the case of ${ℋ}_{2}^{A}$ the objective probability of H is equal to 0.6, whereas in the case of ${ℋ}_{3}^{A}$ the objective probability of T is 0.6. Similar assumptions are made for coin B, namely the agent knows that one of the following three hypotheses is true:


$${ℋ}_{1}^{B}=\left\{\mathrm{CoinBisfair}\right\}$$

$${ℋ}_{2}^{B}=\left\{\mathrm{CoinBfavorsH}\right\}$$

$${ℋ}_{3}^{B}=\left\{\mathrm{CoinBfavorsT}\right\}$$

Let $H_{A}=\left\{{ℋ}_{1}^{A},{ℋ}_{2}^{A},{ℋ}_{3}^{A}\right\}$ and $H_{B}=\left\{{ℋ}_{1}^{B},{ℋ}_{2}^{B},{ℋ}_{3}^{B}\right\}.$

The agent has to decide about her prior probabilities of the above hypotheses. Since there is no direct information supporting one hypothesis over the rest, the agent is likely to subscribe to the principle of indifference (or the maximum entropy principle) according to which:


$$P_{0}({ℋ}_{1}^{A})=P_{0}({ℋ}_{2}^{A})=P_{0}({ℋ}_{3}^{A})=\frac{1}{3}$$

and


$$P_{0}({ℋ}_{1}^{B})=P_{0}({ℋ}_{2}^{B})=P_{0}({ℋ}_{3}^{B})=\frac{1}{3}.$$

Alternatively, the agent might consult her past experience on similar cases (part of her background information) which suggests that it is more often for someone to encounter cases of fair coins than not. In any case, the important thing to notice is that there is no specific information at *t*_0_, hence there is no informational asymmetry between the set of hypotheses concerning coin A and that of coin B.

Let us now calculate the probabilities that the agent assigns to the events/propositions $D_{H}^{A}$ , $D_{T}^{A}$ and $D_{H}^{B}$ , $D_{T}^{B}$ defined as:


$$D_{H}^{A}=\left\{\mathrm{coinAcomesupheads}\right\}=\left\{H_{A}H_{B},H_{A}T_{B}\right\}$$

$$D_{T}^{A}=\left\{\mathrm{coinAcomesuptails}\right\}=\left\{T_{A}H_{B},T_{A}T_{B}\right\}.$$

$$D_{H}^{B}=\left\{\mathrm{coinBcomesupheads}\right\}=\left\{H_{A}H_{B},T_{A}H_{B}\right\}$$

and


$$D_{T}^{B}=\left\{\mathrm{coinBcomesuptails}\right\}=\left\{H_{A}T_{B},T_{A}T_{B}\right\}$$

Using the law of total probability, the agent gets:


$$P_{0}(D_{H}^{A}){=}_{i=1}^{3}P_{0}(D_{H}^{A}|{ℋ}_{i}^{A})P_{0}({ℋ}_{i}^{A})=0.5\times \frac{1}{3}+0.6\times \frac{1}{3}+0.4\times \frac{1}{3}=0.5.$$

In a similar fashion, we obtain,


$$P_{0}(D_{T}^{A})=P_{0}(D_{H}^{B})=P_{0}(D_{T}^{B})=0.5.$$

Let us assume that at time *t > t*_0_ the agent acquires an important piece of specific information for the problem at hand. In particular she is given a set of data, $E_{\infty }^{A}$ , consisting of the results of a very long series of tosses of coin A for which (almost) half of them are H. The following two questions are of interest: (i) how does the agent update her probabilities of $D_{H}^{A}$ , $D_{T}^{A}$ , $D_{H}^{B}$ and $D_{T}^{B}$ in light of the new information, $E_{\infty }^{A}$ about A and (ii) does she end up with a new system of beliefs that violate coherence?

Bayesian conditionalization implies:


$$P_{t}(D_{H}^{A})=P_{0}(D_{H}^{A}|E_{\infty }^{A})=\sum_{i=1}^{3}P_{0}(D_{H}^{A}|{ℋ}_{i}^{A},E_{\infty }^{A})P_{0}({ℋ}_{i}^{A}|E_{\infty }^{A})$$

The Principal Principle implies that (knowledge of the objective chance screens off any admissible evidence):


$$P_{0}(D_{H}^{A}|{ℋ}_{i}^{A},E_{\infty }^{A})=P_{0}(D_{H}^{A}|{ℋ}_{i}^{A}),fori=1,2,3.\;(10)$$

Furthermore, given that the evidence $E_{\infty }^{A}$ is assumed to be arbitrarily large:


$$\begin{array}{l} P_{0}({ℋ}_{1}^{A}\;|\;E_{\infty }^{A})≃1\\ P_{0}({ℋ}_{2}^{A}\;|\;E_{\infty }^{A})≃0\\ P_{0}({ℋ}_{3}^{A}\;|\;E_{\infty }^{A})≃0 \end{array}$$

The above relationships together with ( 10) imply that

$$P_{t}(D_{H}^{A})≅P_{0}(D_{H}^{A}\;|\;H_{1}^{A})P_{0}(H_{1}^{A}\;|\;E_{\infty }^{A})=0.5$$

Similarly,

$$P_{t}(D_{T}^{A})\;=\;0.5.$$

Let us now turn our attention to the events, $D_{H}^{B}$ and $D_{T}^{B}$ (related to coin B) and examine how the acquisition of $E_{\infty }^{A}$ affects their probabilities.

$$P_{t}(D_{H}^{B})=P_{0}(D_{H}^{B}\;|\;E_{\infty }^{A})=\sum_{i=1}^{3}P_{0}(D_{H}^{B}\;|\;H_{i}^{B},E_{\infty }^{A})P_{0}(H_{i}^{B}\;|\;E_{\infty }^{A})$$

As before, PP implies

$$P_{0}(D_{H}^{B}|{ℋ}_{i}^{B},E_{\infty }^{A})=P_{0}(D_{H}^{B}|\;{ℋ}_{i}^{B}),\;fori=1,2,3.$$

Now the crucial question is the following: does the evidence on coin A affect the prior probabilities attached to coin-B related events? Assuming independence between the sets **H**_*A*_ and **H**_*B*_ the answer is negative. Hence,

$$P_{0}({ℋ}_{1}^{B}\;|\;E_{\infty }^{A})=P_{0}({ℋ}_{1}^{B})\;=\frac{1}{3}$$

$$P_{0}({ℋ}_{2}^{B}\;|\;E_{\infty }^{A})=P_{0}({ℋ}_{2}^{B})\;=\frac{1}{3}$$

$$P_{0}({ℋ}_{3}^{B}\;|\;E_{\infty }^{A})=P_{0}({ℋ}_{3}^{B})\;=\frac{1}{3}$$

Finally, the above relationships imply

$$P_{t}(D_{H}^{B})\;=\;P_{t}(D_{T}^{B})=0.5.$$

The important thing to notice is that the agent’s new set of beliefs, *P _t_,* obtained at t (after the specific information $E_{\infty }^{A}$ has arrived) is coherent, that is, it is a proper probability function, i.e. it does not violate the additivity property.

Why did the preceding analysis not produce AA? Put differently, why the acquisition of $E_{\infty }^{A}$ did not produce a system of beliefs having the property

$$P_{t}(D_{H}^{B})\;<\;P_{t}(D_{H}^{A})$$

and

$$P_{t}(D_{T}^{B})\;<\;P_{t}(D_{T}^{A})$$

which would imply an ambiguity averse, probabilistically non-sophisticated agent? The answer is that the analysis presented above implies that the agent follows the MB counterfactual strategy in forming her probability function *P _t_* at t. Indeed, the key point is to focus on how the agent formed her new probability function *P _t_* once she became aware of $E_{\infty }^{A}$ . Specifically, the agent did not use $E_{\infty }^{A}$ as a direct determinant (on a par with background information) of *P _t_.* Instead, she ”traveled back in time” at point *t*_0_ *,* identified *P*_0_ and stuck to those commitments at t ^6^. In this setting, AA would emerge only if the agent abandoned her commitments made at *t*_0_ and assigned different probabilities at t, thus exhibiting dynamic inconsistency.

The preceding analysis implies that the only way for AA to emerge (without causing dynamic inconsistency) is to eliminate *P*_0_. Eliminating *P*_0_ means that at t the agent realizes that she faces a new reality that had not (and could not have) anticipated at t, which obliges her to re-evaluate her full set of probabilistic beliefs (to form a new prior). Such a setting can arise within a large world framework. Teller (1975) gives an example of when such a strategy is required: ”Examples are the wildcatter’s problem of where to drill an oil well and the manufacturer’s problem of how much to produce. The personalists advise the wildcatter and the manufacturer to estimate their initial degrees of belief subjectively for any one such problem, *but if a similar problem arises years later under considerably different circumstances they are advised to make new evaluations rather than to try to conditionalize their old estimates on the vast body of intervening observations of uncertain relevance to the problem*.” (1975, pp. 170, emphasis added). However, Schmeidler’s and Ellsberg’s examples analyzed above are quite different from the wildcatter’s problem. Consider the following question with respect to Schmeidler’s two coin example: is there any reason for the agent to refine her probabilistic judgements at t, because at t she came across a piece of surprising information, that is information that she could not have conceived back at *t*_0_? The answer is negative, because Schmeidler’s two coin example takes place in a small world, where the concept of ”surprising information” is not relevant. As a result, the MB strategy is perfectly feasible; in fact it follows quite naturally!

## 7 Concluding remarks

We conclude by summarizing our main arguments. There are two ways to use specific information, *I _S_,* in constructing the agent’s subjective distribution at t, *P _t_*. The first is to use it as conditioning information, consistent with MB while the second is to use it on a par with any other background information *I _B_,* consistent with CB. There are good reasons, analyzed in detail in section 3, for preferring the first interpretation (MB) over the second (CB). Does the choice of MB over CB have implications for the main issue under scrutiny in the present paper, namely the emergence of AA? The answer is yes. If we choose MB there is no room for AA, since being ambiguity averse in such a framework is equivalent to being dynamically inconsistent. On the other hand, AA can arise under CB because when *I _S_* determines the agent’s subjective distribution, leads to informational asymmetries.

Can we always choose MB over CB? The answer is negative. MB is applicable only in small worlds. In large worlds, MB may not be operational, especially when the agent’s epistemic state at the time that *I _S_* arrives is richer than the one at the time prior to the arrival of *I _S_*, which is the case in large worlds. Hence, AA may be thought of as a (potential) property of large worlds. To this end, the motivation of AA in the form of examples such as Ellsberg’s classic urn-based or Schmeidler’s coin-based, is clearly poor since these examples are textbook cases of small worlds.

## Data availability

All data underlying the results are available as part of the article and no additional source data are required.

## Footnotes

^1^See Maccheroni *et al*. (2006) and Epstein & Schneider (2003) for potential generalizations.

^2^Difference in priors doesn't matter since in the long run, as more evidence is accumulated, these priors "wash out" in the sense that posterior subjective probabilities merge to the objective one.

^3^For example, Hesse (1974) suggests that the agent’s prior probability should include only ”previous background evidence which *does not enter the current calculations*” ( Hesse, 1974 volume pp 53 - emphasis added). She then goes on to explain the nature of the prior probability: ”It is very important to stress that all this (the prior probability) refers only to possibilities, none of which are yet realized at *t*_0_. It refers to what the decision-maker should be prepared to choose in hypothetical circumstances *before more evidence is collected*. It describes the static situation at *t*_0_ in terms of a certain probability distribution satisfying the probability axioms.” (1974, pp. 64, emphasis added).
Teller (1975 same book) argues that in order to be able to conditionalize on *I _S_* (the relevant evidence) an initial probability function, which does not depend on *I _S_*, is required in order for the process of updating beliefs to get started: ”However, for a Bayesian theory fully to characterize the degrees of belief which arise by conditionalization, the theory must specify the belief function from which to start. This initial function is called the prior probability function” (1975, pp. 168-169). Earman (1992) also emphasizes the need of a starting point in order for the conditionalization process to be operational: ”...an agent begins as a *tabula rasa*, chooses her priors, and forever after changes her probabilities only by conditionalization.” (1992, pp. 139-140 original emphasis).

^4^It must be noted that Lewis never gave a precise definition of ”admissibility”. The standard interpretation of this concept is ”that evidence is admissible if it is not relevant to the outcome of the chance event in question. As a rule of thumb, he (Lewis) takes information about the past to be admissible, and information about the future to not be admissible.” ( Meacham, 2007, pp. 18).

^5^ Chihara (1987) found this solution unsatisfactory: ”First note that *K* − { *E*} will probably contain many propositions that imply *E*. ... Thus, to avoid absurdity, it will not be enough to simply delete *E* from *K*: One must also delete all propositions that imply *E*.” In response, Suzuki (2005) tackled this criticism by devising a probabilistic version of AGM ( Alchourron *et al*., 1985) theory. He proceeds in two steps. The first step amounts to deleting the evidential statement *E* from *K*, by adopting a system of classical sentential logic and deleting *E* together with some sentences from *K* so that one *cannot deduce E* in this system. The second step is to adopt what he refers to as ” *ternary-Bayesian confirmation theory”* to construct a new probability measure $P_{K-\{E\}}^{*}$ (from *P*) on *K* − { *E*} under which the problem of old evidence does not arise.

^6^Note that the agent having formed *P*_0_ at *t*_0_ *,* was at an epistemic state of not knowing with certainty that $E_{\infty }^{A}$ has occurred
