# Supplementary material for: Ambiguity aversion, modern Bayesianism and small worlds
Source: Open Res Eur. 2021 Mar 24;1:13. [Version 1] doi: 10.12688/openreseurope.13196.1 (PMC10446098; doi:10.12688/openreseurope.13196.1)
Supplement: Peer review history

# Peer review: Reviewer response for version 1

**DOI:** 10.21956/openreseurope.14266.r27655

**Published:** 2021-10-29

**Authors:** Christoph Kuzmics, Michael Greinecker

**Affiliations:** 1 University of Graz, Graz, Austria

There are decision problems where all uncertainty comes with objective probabilities such as gambling with fair coins and dice. Most real-world decision problems come without natural probabilities, problems in which decision-makers face fundamental uncertainty or, as it is often called, ambiguity. Decision-makers know what their options are and what the possible consequences might be, but not more. The radical subjectivist Bayesian position is that rational decision-makers should be able to quantify all forms of uncertainty - whether they come with natural probabilities or not - in terms of a probability distribution over the states of the world. These probabilities are subjective beliefs, they can even be unique to their owner. L. J. Savage gave the definitive account of the radical subjectivist Bayesian position. Decision-makers choose acts, which are defined as functions from states of the world to consequences. If their preferences satisfy some normatively appealing axioms, they must behave as if they have a numerical index over consequences (``utilities'') and a (uniquely specified) probability assignment on the states of nature such that their preferences can be represented by the expectation of the index with respect to the probability distribution - they are subjective expected utility maximizers.

 In the 1960s, Daniel Ellsberg came up with some thought experiments in which behavior that violates the axioms of Savage seems quite natural and in which people, once the thought experiments were made into actual experiments, indeed often violate the Savage axioms. Importantly, the people whose behavior violated the Savage axioms often stick to their choices when it is explained to them how their choices violate supposed rules of rationality. The Ellsberg experiments gave rise to two literature strands: In one, the actual behavior of people in similar experiments is studied; see Trautmann and Van De Kuilen (2015) for a survey. In the other strand, researchers tried to develop more permissive theories of rationality that allow for the observed behavior in Ellsberg's experiments; see Gilboa and Marinacci (2013) or Machina and Siniscalchi (2014) for surveys. The paper under review is mostly concerned with the latter strand, though the authors also discuss the plausibility of such theories as descriptive theories of human behavior.

 According to a famous argument by Schmeidler (1989), a pioneer of normative theories of decision making that can accommodate the choices from the Ellsberg experiment, the problem with subjective expected utility maximization is that probabilities sweep important differences for a decision-maker under the carpet. Consider a decision-maker who has to bet on whether a coin comes up heads or tails. The decision-maker assigns the same probability to both events. But this could be because they do not know what any potential bias might be or because they have tested the coin extensively and decided it is fair. The assignment of probabilities is the same in both cases and this, argues Schmeidler, shows that probabilities do not contain enough information and a rational individual might treat these situations as different. They might well prefer decisions where they know the probabilities and be averse to fundamental uncertainty - they can be rationally ambiguity averse.

 The authors of the paper under review put the focus on where beliefs, liberally interpreted and possibly including objects other than probability measures, come from. They call such belief-based decision-making Bayesian. ^1^ They draw a distinction between what they term *classical Bayesianism* and *modern Bayesianism*. Classical Bayesians put all previous information into their beliefs, while modern Bayesians only start with, fairly universal, background information in their priors and incorporate all additional information by updating their previous beliefs. We think this is an important point that is not sufficiently in focus in most contemporary work on decision theory: Beliefs have to come from somewhere, and good theories should relate current beliefs to past beliefs and the information learned in between. The modern Bayesian approach is clearly superior to classical Bayesianism on conceptual grounds. However, it requires very detailed and comprehensive models that include all possible sources of information. In small isolated settings (``small worlds'', a term already used by Savage) such as the Ellsberg experiments, this might well be possible. However, in the ``large worlds'' we live in, modern Bayesianism becomes overly demanding. In the language of modern decision theories, one needs to include all possible sources of information one might ever learn in the state space, which will then end up too big to be useful. The authors draw the consequence that ambiguity aversion should be something one may expect to be present in complex real-world decision problems but less so in the original Ellsberg experiments and similar isolated settings.

 We think the paper raises important questions that decision theory ought to focus on, such as the origins of prior beliefs and how they should be interpreted. The conclusion that the maxims of modern Bayesianism are hard to follow in large worlds does not readily imply that commonly used models of ambiguity aversion are good compromises for large worlds. Formal results to that effect would be great contributions in our opinion, and we hope this paper will eventually give rise to such works. To do so, such works should cast the arguments in a more explicit formal setting. For example, it would be desirable to have an explicit representation of the information in *I _B_* and *I _S_*.

 Further more detailed comments:

1. The view that Ellsberg experiments are not the best motivation for ambiguity aversion is also held by others in the literature. For instance, according to Gilboa (2009, p. 136), David Schmeidler is fond of saying that "[r]eal life is not about balls and urns.'' Indeed, Schmeidler's initial research in this area was motivated by what he perceived to be shortcomings of probability distributions as general representations of uncertainty.
2. Similarly, the paper under review points to why ambiguity is best studied for decisions concerning real, large, world events rather than simple artificially created events in the lab. Some such work has begun by, for example, Baillon, Huang, Selim, and Wakker (2018).
3. The present paper partially explains why the two strands of literature, the experimental one and the normative one, developed largely independently. The latter literature does not necessarily aim at understanding how actual people make choices in all settings, acknowledging that people do not always make optimal choices even according to their view of the world, but instead aims at understanding what could be considered rational decision making. The Ellsberg experiments were, thus, just food for thought that provoked a new research direction. The present paper helps to understand why.
4. The survey by Machina and Siniscalchi (2014) focuses on pure theory and is not a good reference for experimental evidence. For experimental evidence, we recommend looking at Trautmann and Van De Kuilen (2015).
5. Savage shows that under his axioms there can be no ambiguity aversion. The first line of the second paragraph on page 4 should, therefore, be stated differently.
6. There is extensive literature on the relationship between dynamic consistency (usually in combination with a version of consequentialism) and ambiguity aversion. The authors mention that ambiguity aversion leads to dynamic inconsistencies but do not quote from the relevant literature. A readable introduction to the topic is given by the debate between Al-Najjar and Weinstein (2009) and Siniscalchi (2009).

^1^Usually, the term Bayesian is used more narrowly. In particular, in decision theory, Bayesianism is often identified with the view that rational behavior maximizes subjective expected utility. However, the statistician I. J. Good counted 46656 possible varieties of Bayesianism in a letter to the editor of *The American Statistician* in 1971.

Is the study design appropriate and does the work have academic merit?

Yes

Is the work clearly and accurately presented and does it cite the current literature?

Partly

If applicable, is the statistical analysis and its interpretation appropriate?

Not applicable

Are all the source data underlying the results available to ensure full reproducibility?

No source data required

Are the conclusions drawn adequately supported by the results?

Yes

Are sufficient details of methods and analysis provided to allow replication by others?

Yes

Reviewer Expertise:

Decision Theory, Game Theory, Statistics

We confirm that we have read this submission and believe that we have an appropriate level of expertise to confirm that it is of an acceptable scientific standard.

## References

[1] : THE AMBIGUITY AVERSION LITERATURE: A CRITICAL ASSESSMENT. *Economics and Philosophy* .2009;25(3) : 10.1017/S026626710999023X 249-284 10.1017/S026626710999023X

[2] : Measuring Ambiguity Attitudes for All (Natural) Events. *Econometrica* .2018;86(5) : 10.3982/ECTA14370 1839-1858 10.3982/ECTA14370

[3] : Theory of Decision under Uncertainty.2009; 10.1017/CBO9780511840203 10.1017/CBO9780511840203

[4] : Ambiguity and the Bayesian Paradigm.2013; 10.1017/CBO9781139060011.008 179-242 10.1017/CBO9781139060011.008

[5] : Ambiguity and Ambiguity Aversion.1: 10.1016/B978-0-444-53685-3.00013-1 729-807 10.1016/B978-0-444-53685-3.00013-1

[6] : Subjective Probability and Expected Utility without Additivity. *Econometrica* .1989;57(3) : 10.2307/1911053 10.2307/1911053

[7] : TWO OUT OF THREE AIN'T BAD: A COMMENT ON “THE AMBIGUITY AVERSION LITERATURE: A CRITICAL ASSESSMENT”. *Economics and Philosophy* .2009;25(3) : 10.1017/S0266267109990277 335-356 10.1017/S0266267109990277

[8] : Ambiguity Attitudes.2015; 10.1002/9781118468333.ch3 89-116 10.1002/9781118468333.ch3

# Peer review: Reviewer response for version 1

**DOI:** 10.21956/openreseurope.14266.r27354

**Published:** 2021-08-18

**Authors:** Gerit Pfuhl

**Affiliations:** 1 Human Factor in High Risk Environments, UiT The Arctic University of Norway, Tromso, Norway

Pittis *et al*. derived, from applying modern Bayesianism, that a rational agent cannot be ambiguity averse (AA) in a small world.

 They build up to their conclusion by using the small world two coin example (Schmeidler's) and show that a rational agent of modern Bayesianism is dynamically consistent and hence ambiguity neutral. The agent is consistent because new (specific) information is not changing the background information, and does not violate the additivity property either. 

 Behaviourally, AA has been reported in small world contexts like the Ellsberg two-urn paradox. However, as Trautmann and van de Kuilen (2015) reviewed, AA may depend on the elicitation method and is not found for unlikely events. This might refer to the discussion whether event A is included in the specific information (and hence relevant for the outcome and the Principal Principle cannot be applied) or not.

 As the authors nicely show, modern Bayesianism (employing a counterfactual strategy and using hypothetical priors) is only feasible in small worlds as large worlds run into coherence problems (one can hold conflicting beliefs, see e.g., Caie 2013 or Tarnowski 2020 "Is having contradictory beliefs possible? Discussion and critique of arguments for the psychological principle of non-contradiction").

 In a coherent belief system a modern Bayesian agent cannot be ambiguity averse. But since coherence is not given in large worlds, nor necessarily in all small worlds (e.g., coin task but participant conspicuous about task purpose) behaviourally one may still find AA. The results could potentially explain why AA (behaviorally) shows low reliability (e.g., test-retest) and is more a state, not a trait (see also Carnap "traits") or said differently indicating that participants are better described as agents using classical Bayesianism not modern Bayesianism. 

 The article should stimulate further research into Simon's "scissor blades" and identify the boundaries of human rationality. 

 A few minor issues

1) The authors introduce all symbols and abbreviations but D (data, see page 5).
Page 8 second paragraph has three questions, though it is clear what is meant by "MB responds negatively to the first question and positively to the second".
page 9 "difference that the role that I_s plays" - could it be written as "different role that I_s plays" ?
page 9 "the agent cannot condition on A since it has already determined her probability function" - I would replace it with she.
page 10 "the agent had assign" <- ed missing, should be "had assigned".
page 10 "corresponding to I_B and I_B, respectively," should be I_B and I_S?
page 13: "MB and CB refer to the agent's strategy or course of action in forming her probability function at GB and LB refer to the agent's epistemic status ..." - I am wondering whether the sentence should have a "and" before / instead of at "GB and LB refer to ..."
8) page 13 Ellsber's <- g missing.

Is the study design appropriate and does the work have academic merit?

Yes

Is the work clearly and accurately presented and does it cite the current literature?

Yes

If applicable, is the statistical analysis and its interpretation appropriate?

Not applicable

Are all the source data underlying the results available to ensure full reproducibility?

Yes

Are the conclusions drawn adequately supported by the results?

Yes

Are sufficient details of methods and analysis provided to allow replication by others?

Yes

Reviewer Expertise:

Decision-making under uncertainty, cognitive biases, human rationality

I confirm that I have read this submission and believe that I have an appropriate level of expertise to confirm that it is of an acceptable scientific standard.

## References

[1] : Ambiguity Attitudes.2015; 10.1002/9781118468333.ch3 89-116 10.1002/9781118468333.ch3

[2] : Rational Probabilistic Incoherence. *Philosophical Review* .2013;122(4) : 10.1215/00318108-2315288 527-575 10.1215/00318108-2315288

[3] : Is having contradictory beliefs possible? Discussion and critique of arguments for the Psychological Principle of Non-Contradiction. 2020; 10.26333/stsen.xxxi.04 10.26333/stsen.xxxi.04
